# Peritoneal dialysis catheter salvage performed by nephrologists in tunnel exposure management

**DOI:** 10.1186/s12882-022-02804-9

**Published:** 2022-05-05

**Authors:** Seok Hui Kang, A. Young Kim, Jun Young Do

**Affiliations:** Division of Nephrology, Department of Internal Medicine, College of Medicine, Yeungnam University, 170 Hyeonchung-ro, Nam-gu, Daegu, 42415 Republic of Korea

**Keywords:** Catheter salvage, Peritoneal dialysis, Revision, Tunnel exposure

## Abstract

**Background:**

Tunnel exposure, a non-infectious complication, is a rare finding in peritoneal dialysis (PD) patients, which has been described in some case reports. Our study aimed to present catheter salvage therapy using a revision procedure of tunnel exposure by nephrologists.

**Methods:**

Our retrospective study was conducted between July 1998 and October 2021. We identified all PD patients with tunnel exposure from a database of a tertiary medical center. Tunnel exposure was diagnosed following gross inspection by clinicians during outpatient consultations. We attempted revision with partial external cuff shaving and creating a new tunnel without catheter change.

**Results:**

Fourteen cases in 12 patients were diagnosed as tunnel exposure. The median age at presentation of tunnel exposure was 51 years. Eleven patients underwent revision, and the PD catheter was removed in one patient. The patients who underwent revision were followed up for 6 months. The catheter salvage rate was 72.7%.

**Conclusions:**

The present study demonstrated that catheter revision performed by nephrologists could be a valuable alternative for original catheter salvage before considering catheter removal in tunnel exposure management.

## Introduction

Although peritoneal dialysis (PD) has many merits compared to hemodialysis (HD), it has been underutilized in many countries [1, 2]. A previous study suggested that this problem could be improved by PD catheter insertion performed by nephrologists [3]. PD catheter insertion by nephrologists can improve the utilization of PD, but various complications requiring surgical intervention after PD catheter insertion are a hurdle. Therefore, the proper recognition and treatment of PD catheter-related complications, which may require surgical intervention, would be indirectly useful in reducing the barrier to induce PD catheter insertion by nephrologists. Furthermore, this could lead to the maintenance of long-term PD and decrease the transition from PD to HD.

PD catheter-related complications represent an important hurdle in maintaining long-term PD. They include non-infectious complications, such as ultrafiltration failure and migration, and infectious complications, such as exit-site and tunnel infection. Tunnel exposure, a non-infectious complication, is a rare finding in PD patients, which has been described in some case reports [4–6]. Therefore, information regarding the nature and proper treatment of tunnel exposure is relatively limited, leading to insufficient or improper treatment of this condition. Finally, this process results in PD catheter removal regardless of the medical requirements. Therefore, our study aimed to present catheter salvage therapy using a revision procedure of tunnel exposure by nephrologists.

## Materials and methods

Our retrospective study was conducted between July 1998 and October 2021. We identified all PD patients with tunnel exposure from a database of a tertiary medical center. This study received the ethical approval of the Institutional Review Board of Yeungnam University Hospital and was conducted following the principles of the World Medical Association Declaration of Helsinki (approval number: 2021–11-048). All PD catheters had a double-cuff swan neck design with silicone material and were inserted by nephrologists using one of the following two methods [7]: (1) PD catheter insertion using the trocar method was performed at the midline below the umbilicus, and the internal cuff was positioned at the linea alba. (2) PD catheter insertion by surgical method was performed at the para-median beside the umbilicus, and the internal cuff was positioned within the rectus muscle. Dressing changes with exit site cleaning and antibiotic ointment were performed at least twice weekly. Exit-site cleaning was performed using povidone iodine or chlorhexidine and mupirocin ointment. In addition, our center routinely evaluated whether each participant was a nasal methicillin-resistant *Staphylococcus aureus* carrier, but additional interventions were not performed for carriers.

Tunnel exposure was diagnosed following gross inspection and palpation of the lesions by clinicians during outpatient consultation. Tunnel exposure was defined as gross exposure of the PD catheter segment through the eroded skin above the subcutaneous tunnel, regardless of the length or the extent of infection. Cases with grossly external or internal cuff extrusion were excluded from our presentation.

**Fig. 1:**
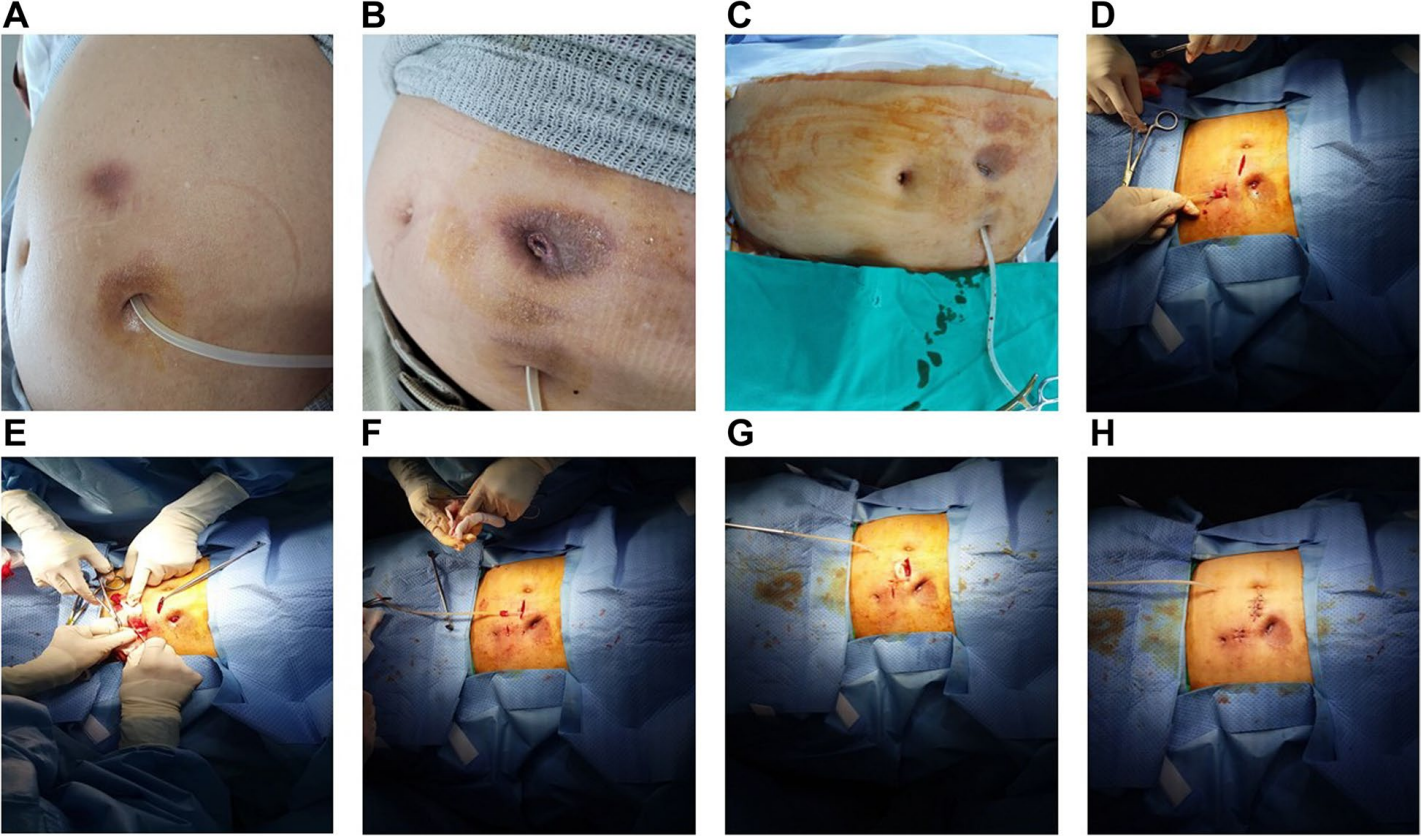
Catheter revision procedure in a patient who underwent catheter insertion using paramedian approach. **A** and **B** Skin changes before and after tunnel exposure. **C** and **D** Under local anesthesia, two skin incisions were performed at the superior superficial and deep cuff. **E** Cuff shaving of the superficial cuff. **F**, **G**, and **H** Formation of a new exit and tunnel at midline level

Catheter revision was primarily considered in patients with tunnel exposure at our center. Detailed revision procedures are shown in Fig. 1 as well as a previous study [8]. Briefly, the revision was performed by two nephrologists in an operating or PD room. The pericatheter and the incision area were sterilized using povidone iodine. Local anesthesia was administered using lidocaine. The previous incision and external cuff sites were incised, and the external cuff was dissected using electrocautery. The operator carefully inspected and palpated the track of the catheter segment between the internal cuff and the exit site. If the patient showed no culture growth despite evidence of tunnel infection or an incidentally identified abscess during revision, an additional culture was performed using discharge or pus from the infected lesions. The internal cuff was not dissected in cases that underwent revision surgery only. The internal cuff for eleven cases, inserted using the trocar method, was positioned above the linea alba, and the involvement of the internal cuff could be easily evaluated without further dissection. The internal cuff for one case, inserted using the surgical method, was positioned within the rectus muscle, but dissection was not performed. Internal cuff involvement was evaluated by inspection and palpation of the adjacent tissue around the internal cuff. For the case inserted using the surgical method, the internal cuff tightly adhered to the adjacent tissue, and the tissue around the internal cuff did not have discharge, pus, or tissue defects. Therefore, we concluded that the case did not involve the internal cuff. The infected and/or nectrotic debris were grossly identified and removed entirely. The tunnel site was sterilized using povidone iodine and hydrogen peroxide. The whole catheter segment was sterilized by covering it with povidone iodine-containing gauze for more than 10 min. Partial cuff shaving was performed in all patients who underwent revision, and the external cuff was shaved until all the tissues adherent to the cuff were completely removed. In addition, we performed re-sterilization using povidone iodine. A new subcutaneous tunnel was created at a contralateral position or away from the previous course, and a new transfer set was inserted.

Tunnel or exit site infection was defined as purulent discharge with or without erythema adjacent to the tunnel exposure or exit site. If the results of culture were available, therapeutic antibiotics were administered according to the results of culture. If the culture was not performed owing to the lack of evidence of infection, cephalosporin or ciprofloxacin were used as prophylactic antibiotics before the revision operation. Relevant antibiotics were maintained until wound healing was achieved after the revision. PD was restarted from the day of the operation without a break time after revision.

Data were analyzed using the statistical software IBM SPSS Statistics (version 25; SPSS Inc., Chicago, IL, USA). Categorical variables are expressed as counts (percentages). Continuous variables were evaluated for the distributional assumption of normality using Kolmogorov–Smirnov test. Continuous variables are expressed as mean ± standard deviation for those with normal distribution and median (interquartile range) for those with non-normal distribution.

## Results

In our center, 1259 patients underwent PD catheter insertion between July 1998 and October 2021. Fourteen cases in 12 patients were diagnosed as tunnel exposure (Table 1). The incidence of tunnel exposure was approximately 1%. Two patients (Patient No. 11 and 12) had two events each of tunnel exposure. The age at presentation of tunnel exposure was 51 (22) years. The interval between PD catheter insertion and presentation of tunnel exposure was 48 (57) months. Female sex was the predominant sex (7 patients, 58.3%). The underlying causes of the end-stage renal disease were glomerulonephritis (5 patients, 41.7%), diabetes mellitus (4 patients, 33.3%), and hypertension (3 patients, 25%). PD catheter insertion using the trocar method was performed in all patients except one. All patients underwent continuous ambulatory PD. Nine patients received 2 L of dialysate per exchange, 1.5 L for one patient, and 2.5 L for two patients. The number of exchanges per day was two exchanges for one patient, three exchanges for two patients, and four exchanges for nine patients. The median body mass index value was 20.3 (3.9) kg/m^2^. Two patients had a history of abdominal surgery (subtotal gastrectomy for patient no. 3 and hysterectomy for patient no. 8). One patient (patient no. 2) received steroids. There were no patients with fluid leakage, the use of other immunosuppressants, polycystic kidney disease, or a histroy of trauma to the abdominal wall other than surgery.

**Table 1:** Participants’ clinical characteristics and outcomes

PT No	Age	Sex	DS	PDCI method	Months between PDCI and TE	Culture	ESI/TI	Intervention for TE	FU duration after REV (M)	Control of ESI/TI after REV	Outcomes after revision
1	37	F	HTN	Trocar	17	NG	Yes	REV	35	Yes	KT with functioning PDC
2	25	F	GN	Trocar	42	MSSA	Yes	REV	18	Yes	PDC removal d/t recurrent ESI/TI at new tunnel
3	69	M	CGN	Trocar	122	NG	No	REV	31	Yes	Maintenance of PD without problem
4	57	M	DM	Trocar	54	EC	Yes	REV	6	Yes	Patient death with PDC without problem
5	52	F	DM	Trocar	128	NG	No	PDC removal	–	–	–
6	79	M	DM	Surgical	55	MSSA	Yes	REV	3	Yes	Maintenance of PD without problem
7	60	M	HTN	Trocar	98	NG	No	REV	35	Yes	PDC removal d/t recurrent peritonitis
8	43	F	GN	Trocar	48	MRSA	Yes	REV	3	Yes	KT with functioning PDC without problem
9	30	F	GN	Trocar	47	MSSA	Yes	REV	3	No	PDC removal d/t recurrent ESI/TI at TE site
10	51	F	DM	Trocar	16	PS	Yes	REV	1	Yes	Transfer without problem
11	37	M	GN	Trocar	48	PS	Yes	REV	24	No	Re-revision d/t recurrent ESI/TI and PDC removal d/t recurrent ESI/TI despite 2 revisions
12	50	F	HTN	Trocar	27	MSSA	Yes	REV	3	No	PDC removal d/t recurrent TE

Abbreviations: *PT No* patients number, *F* female, *M* male, *DS* underlying disease of end-stage renal disease, *HTN* hypertension, *GN* glomerulonephritis, *DM* diabetes mellitus, *PDCI* peritoneal dialysis catheter insertion, *TE* tunnel extrusion, *NG* no growth, *MSSA* methicillin-sensitive *Staphylococcus aureus*, *EC Escherichia coli*, *MRSA* methicillin-resistant *Staphylococcus aureus*, *PS Pseudomonas aeruginosa*, *ESI/TI* exit-site and/or tunnel infection, *REV* revision, *PDC* peritoneal dialysis catheter, *FU* follow-up, *KT* kidney transplantation, *PD* peritoneal dialysis, *d/t* due to

Nine patients had signs or symptoms of infection at the presentation of tunnel exposure, and the causative organisms were methicillin-sensitive *Staphylococcus aureus* (4 patients), *Pseudomonas aeruginosa* (2 patients), methicillin-resistant *Staphylococcus aureus* (1 patient), *Escherichia coli* (1 patient), and no growth (1 patient). Eleven patients underwent revision, and the PD catheter was removed in one patient. For the exposure site, two patients received povidone-soaked dressing for 14 days. The other patients had a simple dressing after povidone cleansing of the adjacent opening. The dressing was changed at the exposure site daily until the wound healed. The patients who underwent revision were followed up for 6 (28) months. Eight patients who had undergone revision did not have additional complications associated with the previous tunnel exposure and the revision procedure (72.7%). PD catheter removal after the revision was performed in 3 patients due to recurrent infection at the past tunnel exposure site or recurrent tunnel exposure. The median duration of antibiotics use after the revision was 14 (10) days (patient no. 1, third generation cephalosporin and ciprofloxacin for 14 days; patient no. 2, first generation cephalosporin for 10 days; patient no. 3, third generation cephalosporin for 6 days; patient no. 4, ceftazidime and aminoglycoside for 14 days; patient no. 5, third generation cephalosporin for 9 days and ciprofloxacin for 7 days; patient no. 6, ciprofloxacin for 14 days; patient no. 7, third generation cephalosporin for 7 days; patient no. 8, ceftazidime and ciprofloxacin for 28 days; patient no. 9, vancomycin for 2 months; patient no. 10, ceftazidime and vancomycin for 21 days; patient no. 11, ceftazidime and aminoglycoside for 21 days; patient no. 12, third generation cephalosporin for 7 days and first generation cephalosporin for 7 days).

## Discussion

Tunnel exposure is a rare PD catheter-related complication. Our study included the largest number of cases with PD catheters complicated by tunnel exposure to the best of our knowledge. In all except one patient presenting with extensive infection, we attempted revision with partial external cuff shaving and creating a new tunnel without catheter change. The catheter salvage rate was 72.7%.

A previous study presented a patient with tunnel exposure caused by infection, and the catheter was removed due to severe infection [5]. Two studies showed two patients with tunnel exposure without a definite infection. One of the two cases was treated using excision of skin and subcutaneous tissues around the exposure site and a simple suture without manipulating the external cuff and the exit site [4, 6]. Figure 1A and B show typical skin changes around the tunnel before and after tunnel exposure, including hyperpigmented and indurated skin lesions. Figure 1 presents a patient who had developed tunnel exposure three months after the presentation of the skin change. Our study did not define whether the primary cause of tunnel exposure in all cases was tunnel infection or pressure of PD catheter against soft tissues, regardless of infection. However, we treated 11 subjects using the same revision procedures, and 3 patients without infection signs did not have additional complications after revision.

Tunnel exposure can be developed by sustained pressure of the PD catheter to the surrounding tissues and/or posterior tunnel infection. Therefore, prevention or treatment of the exit-site and/or tunnel infection and decreasing the pressure on the subcutaneous tissues/skin through the tunnel may be an important step in preventing tunnel exposure. First, to decrease the exit site and/or tunnel infection, recent guidelines recommend cleaning and/or topical application of antibiotic ointment to the exit site, frequent inspection of exit site, and proper treatment using antibiotics for infection symptoms or signs [9]. Second, decreasing the pressure on the subcutaneous tissues/skin through the tunnel would help prevent tunnel exposure. The catheter segment between the two cuffs can be prone to the pressure of the subcutaneous tissues/skin for the two cuffs swan neck catheter. Therefore, it can be useful to embed sufficient subcutaneous tissue above the catheter segment between two cuffs, including a bending site in especially thin patients without sufficient subcutaneous tissues. A previous study evaluated the risk factors for abdominal wall complications, such as peritoneal leak or hernia, in PD patients, and these factors may be associated with tunnel exposure as a complication [10]. Our cases were not highly prevalent for these risk factors, but interpretation of our results should be carefully performed owing to the limitations of the small sample size and retrospective study design.

Tunnel exposure to the PD catheter is a rare complication. There are few studies regarding the proper management of tunnel exposure or alternative treatment options owing to the rarity of this complication. Most centers may perform catheter removal and reinsertion of a new catheter for this complication, which is the standard treatment for tunnel exposure. However, the procedure is relatively time-consuming, and psychological resistance may exist in some patients. Some patients may want to transfer to HD, as they misunderstand tunnel exposure as a severe complication requiring surgery. Furthermore, catheter replacement could require a new incision for the new PD catheter insertion, which may waste a new PD catheter reinsertion site in the absolute indication of PD catheter removal. In addition, the procedure needs to be performed in the operating room owing to the exposure of the intraperitoneal cavity. Our study is meaningful in presenting an alternative or bridging method for treating tunnel exposure in the PD catheters. Nevertheless, our procedure does not completely exclude infectious materials in the original catheter segment, which is associated with complications of infection after the procedure. Although our patients who underwent revision used fully original catheters, partial replacement of the catheter segment, as described in previous studies, could be another option for decreasing recurrent infection after revision for tunnel exposure [11–13]. Patients with a high risk for recurrent infection should consider catheter removal and reinsertion; however, those with low or moderate risk for recurrent infection may consider revision with or without partial replacement of the catheter as an alternative option for tunnel exposure. Although there were no definite guidelines that could have been used to define a high risk of recurrent infection after revision for tunnel exposure, those with close distance between the exposed/infectious lesion and internal cuff or new tunnel, infection by invasive organisms, such as gram-negative organism or fungus, simultaneous peritonitis, and extensive infection can be considered at high risk of recurrent infection after revision operation. If there is no evidence of infection, excision of the adjacent tissue around the exposure and suturing without manipulation of the external cuff may be sufficient. In addition, for all cases, the use of antibiotics is essential during the period before and after revision or removal.

Tunnel infections can precede or coexist with tunnel exposure. Early identification and treatment for infections at the skin or subcutaneous tissue around the catheter can be helpful in preventing tunnel exposure or decreasing complications after revision. Furthermore, some preventive interventions, such as glycemic control, mupirocin application for the nasal carriage of *Staphylococcus aureus*, exit-site care in a clean environment, and avoidance of injecting insulin or erythropoiesis-stimulating agents around the catheter could be recommended in clinical practice [9].

Our study has some limitations. Our study was of a retrospective study design, and data were collected at a single center over a long period owing to the rarity of this complication. In particular, data collection over a long period can be associated with differences in treating physicians, protocols for exit-site care, or the amount of clinical data collected from each case, which would lead to performance or selection biases. Therefore, the results of our study should be carefully interpreted, and the generalizability of our results is limited. In addition, our study did not compare various interventions according to the status of tunnel exposure. Future large scale studies are warranted to overcome these limitations.

In conclusion, the present study demonstrated that catheter revision performed by nephrologists could be a valuable alternative for original catheter salvage before considering catheter removal in tunnel exposure management.

## Data Availability

The datasets generated and/or analysed during the current study are not publicly available due to limitations of ethical approval involving the patient data and anonymity but are available from the corresponding author on reasonable request.

## Abbreviations

HD: Hemodialysis
PD: Peritoneal dialysis
